# Re-epithelialization and immune cell behaviour in an *ex vivo* human skin model

**DOI:** 10.1038/s41598-019-56847-4

**Published:** 2020-01-08

**Authors:** Ana Rakita, Nenad Nikolić, Michael Mildner, Johannes Matiasek, Adelheid Elbe-Bürger

**Affiliations:** 1 Department of Dermatology, Medical University of Vienna, Vienna, Austria; 2 Department of Plastic, Aesthetic and Reconstructive Surgery, St. Josef Hospital, Vienna, Austria

**Keywords:** Applied immunology, Immunological techniques

## Abstract

A large body of literature is available on wound healing in humans. Nonetheless, a standardized *ex vivo* wound model without disruption of the dermal compartment has not been put forward with compelling justification. Here, we present a novel wound model based on application of negative pressure and its effects for epidermal regeneration and immune cell behaviour. Importantly, the basement membrane remained intact after blister roof removal and keratinocytes were absent in the wounded area. Upon six days of culture, the wound was covered with one to three-cell thick K14^+^Ki67^+^ keratinocyte layers, indicating that proliferation and migration were involved in wound closure. After eight to twelve days, a multi-layered epidermis was formed expressing epidermal differentiation markers (K10, filaggrin, DSG-1, CDSN). Investigations about immune cell-specific manners revealed more T cells in the blister roof epidermis compared to normal epidermis. We identified several cell populations in blister roof epidermis and suction blister fluid that are absent in normal epidermis which correlated with their decrease in the dermis, indicating a dermal efflux upon negative pressure. Together, our model recapitulates the main features of epithelial wound regeneration, and can be applied for testing wound healing therapies and investigating underlying mechanisms.

## Introduction

Expanding knowledge about wound healing mechanisms and research regarding this topic is necessary to improve existing techniques of wound treatment. To fully restore epidermal barrier function, a wound requires regeneration of the epidermis through wound re-epithelialization, where keratinocytes migrate and differentiate to complete this process^1^. Keratin 14 (K14) and K5 expressed in mitotically active basal layer cells^2,3^ are substituted by K1 and K10 when entering the terminal differentiation program^4^. To obtain flexibility to migrate during the epithelialization process, keratinocytes must detach from the basement membrane as well as the neighbouring cells. Hemidesmosomes and desmosomes loosen, allowing migration of keratinocytes from the wound edges over the wounded area^5,6^.

Numerous *in vivo* and *ex vivo* animal skin wound models have been established. Animal models have the great advantage of manifesting the complexity of an entire organ and its interactions with other organs. However, besides ethical concerns related to the use of animals (worldwide accepted 3 R principle (Replacement, Reduction, Refinement)), each animal model shows non-negligible limitations such as thickness of the skin, different primary healing mechanisms (e.g., contraction in mice and rats), and diverse duration of wound healing^7^. Thus, due to anatomical and physiological differences, no animal model could ever fit all needs required for human wound research making it often difficult to translate basic and preclinical data into the clinic. Such differences underline the need to further develop and adapt existing human wound healing models and to develop other, even more representative and reliable models^7,8^.

To study re-epithelialization and wound healing in human skin, different *ex vivo* human skin models have been established. Incisional *ex vivo* human skin wounds created with a scalpel or partial-thickness wounds initiated with a small biopsy punch were shown to re-epithelialize^9,10^. Unlike these models, where the basement membrane and partially the dermal structure are disrupted, several dermal-epidermal separation methods have been established providing a better basis to study re-epithelialization^11^. However, none of the methods is appropriate for all purposes and several research questions require another separation technique. Simplicity of separation by inducing heat is accompanied by its damaging influence on both epidermis and dermis^12^. Chemical reagents disturb the electrolyte cellular equilibrium, or in case of enzymes, kill important components^13,14^. Mechanical forces used so far to separate epidermis from dermis include mechanical stretching and suction, which have the advantage of not inducing any chemical changes concerning epidermis, dermis and basement membrane^11^. A suction device using negative pressure to produce blisters on human skin *in vivo* has been reported more than five decades ago. The blister roof consisted of a viable epidermis including the keratinocyte basal cell layer while leaving the basement membrane intact^15,16^. A modification of the suction device by heating revealed a faster formation of blisters allowing keratinocytes and epidermal Langerhans cells (LCs) to preserve their shape and viability. Today, suction blisters find their use in various fields of dermatological research, mainly to create standardized wound healing models and to study physiological, morphological and pharmacological phenomena^11^. Müller and colleagues used blister fluid from healthy individuals and conducted a comparative proteomic study using immunodepletion and isobaric tags for relative and absolute quantitation (iTRAQ)^17^. In another study, effects of a topically applied calcineurin inhibitor and corticosteroids have been investigated on LCs using blister roofs from healthy and atopic dermatitis patients for evaluation^18^. Recently, Polak and colleagues used suction blister fluid of allergic patients injected intradermally with grass pollen extracts and tested the role of neutrophils in IgE-mediated allergy^19^. Research in animals and humans using the suction blister device so far was conducted *in vivo* only.

We have successfully utilized the device on human *ex vivo* skin and present that its application is comparable to *in vivo* experiments, and is a standardized, consistent and reproducible model, recapitulating the main features of epidermal wound regeneration.

## Results

### Keratinocytes re-epithelialize the wound bed upon culture

**Figure 1:**
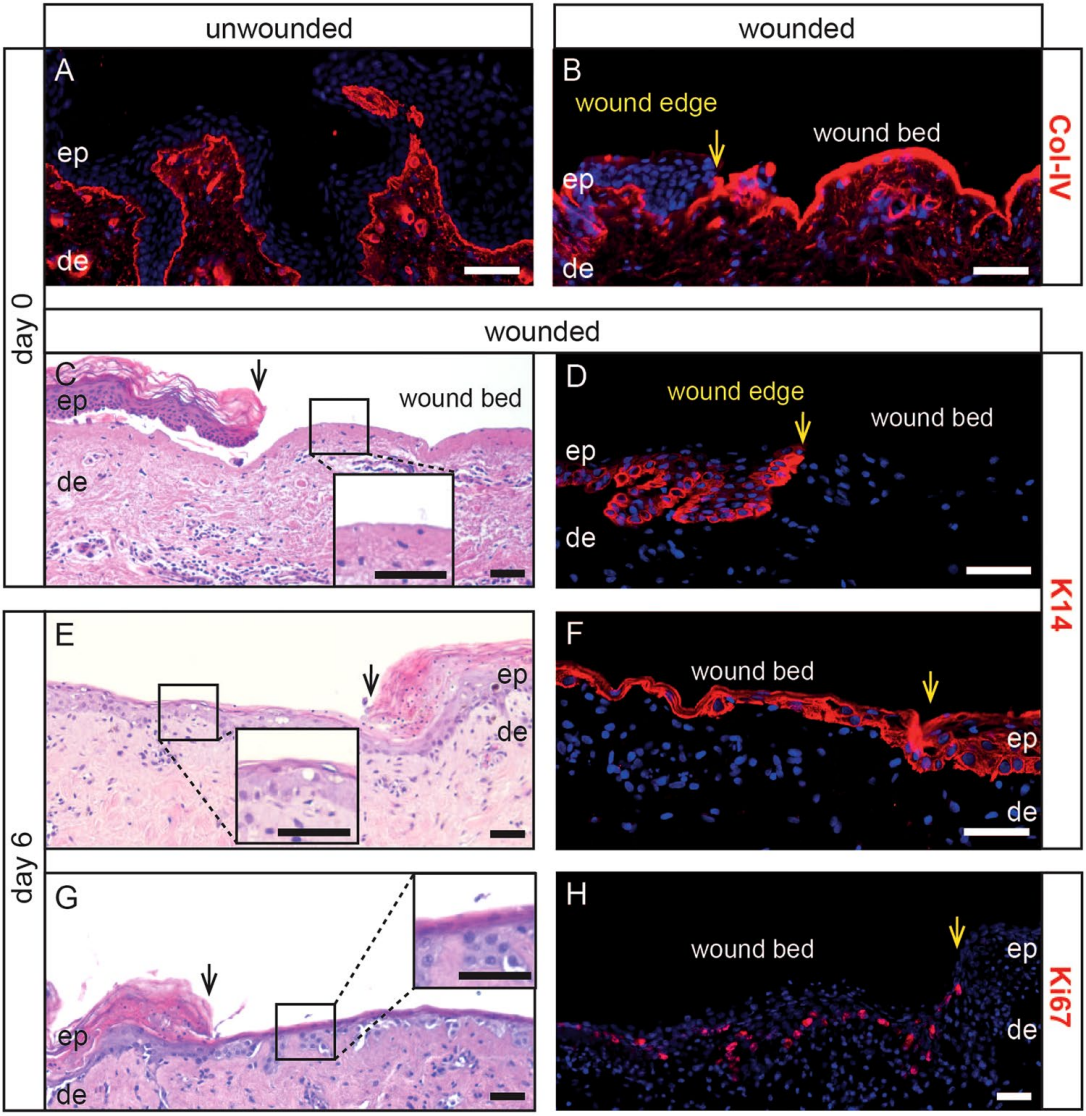
Re-epithelialization of *ex vivo*-induced epidermal wounds upon culture. Type IV Collagen (Col-IV) staining in cryostat sections of freshly isolated unwounded (**A**) and suction blister wounded skin (**B**). Hematoxylin and eosin (H&E) stained paraffin sections show epidermis and dermis of a freshly wounded (**C**, insert) and 6 day-cultured wounded biopsy (**E**,**G**, inserts). Immunofluorescent labeling demonstrates that K14^+^ keratinocytes are present in the unwounded epidermis but absent in the wound bed before culture (day 0; **D**). The wound was closed by a one to three cell thick K14^+^ keratinocyte layer on day 6 upon culture (**F**). Several keratinocytes express the proliferation marker Ki67 above the initial wound bed (**H**). Black and yellow arrows indicate the wound edge. Nuclei are visualized with 4′,6-diamidino-2-phenylindole (DAPI, blue). One representative of six (**C,E,G**) or three (**A,B,D,F,H**) experiments is demonstrated. ep, epidermis; de, dermis. Scale bars = 50 µm.

The well established *in vivo* suction blister model^20^ is often used in tissue serum research in the pharmaceutical and cosmetic research fields^21^ to investigate the blister fluid^17^, blister fluid cells and blister roofs from healthy and diseased skin^18,19^ but thus far, to our knowledge, was never utilized *in vitro*. When applying this method on *ex vivo* human skin, blisters formed repeatedly much later (blisters form upon 6–8 h) as compared to the *in vivo* situation (1–2.5 h). Type IV collagen staining was found on the base of the blister and was comparable to the staining pattern in the unwounded area on the same section (Fig. 1A,B). Thus, the basement membrane keeps its integrity after blister roof removal, similar to previous observations *in vivo*^22^. Besides measuring drug concentrations in various parts of the skin^16^, the suction blistering model was employed to study wound healing *in vivo*. It has been shown that after blister roof removal, clean wounds can heal without scar formation^23^. Also a long-term course of epidermal regeneration in healthy humans was studied with this model^24^. To test whether wounds induced by *ex vivo* blistering are similarly able to re-epithelialize, punched biopsies upon removal of the blister roof were analysed at several time-points of culture. Compared to fresh wounds (Fig. 1C), wounds repeatedly and completely re-epithelialized on day 6 of culture (Fig. 1E,G). Immediately after injury, K14^+^ keratinocytes were present in non-wounded epidermis but absent in the wound bed (Fig. 1D). A strong K14 staining was observed along the initial wound bed on day 6 of culture, indicating and confirming a complete wound closure (Fig. 1F). In line with this, we observed up to 20% Ki67^+^ keratinocytes along the initial wound bed on day 6, while they were sparse in the basal layer of unwounded human epidermis in the same section (Fig. 1H).

### A multi-layered epidermis expressing epidermal differentiation markers forms after wound closure

**Figure 2:**
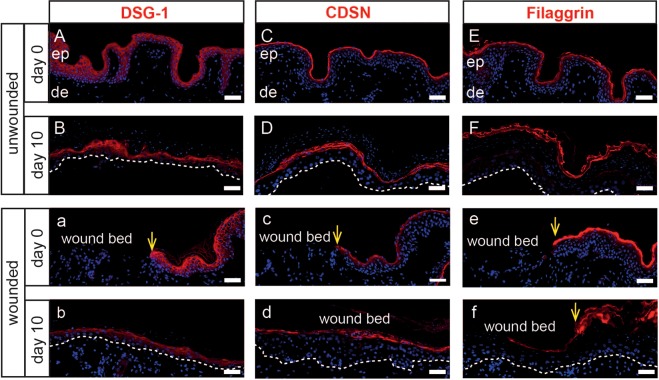
Expression of epidermal differentiation proteins in re-epithelialized skin. As compared to normal skin (**A,C**), expression of Desmoglein (DSG-1) and Corneodesmosin (CDSN) is observed in lower epidermal layers at day 10 of culture in unwounded skin (**B,D**). They appear in low epidermal layers above the initial wound bed in wounded skin (b,d). Filaggrin is expressed at the beginning of the culture and at day 10 in untreated cultured biopsies (**E,F**). A layer, expressing filaggrin was not present in wounded skin (e) and emerged above the initial wound bed at 10 day after wounding (f). Yellow arrows indicate the wound edges. White dashed lines indicate the dermal-epidermal boundary. Nuclei are visualized with DAPI. One representative of three experiments is demonstrated. ep, epidermis; de, dermis. Scale bars = 50 µm.

We next tested whether keratinocytes upon wound closure can also differentiate and form multiple epidermal layers upon prolonged culture. Indeed, several K14^+^ keratinocyte layers were observed upon 8 days of culture above the initial wound bed (Fig. S1A,C). During the continued culture period of 12 days, multiple K14^+^ layers have formed (Fig. S1B,D). In normal, unwounded skin, K10 is exclusively localized in suprabasal keratinocytes (Rakita, unpublished observation). In wounded skin, a few K10^+^ basal keratinocytes, in particular at the wound edge, were observed in addition to the conventional staining pattern in suprabasal keratinocytes at day 8 of culture (Fig. S1E). K10 expression appeared in all layers except the basal layer at later time points (Fig. S1F). Next, expression kinetics of particular epidermal differentiation markers in unwounded skin and blister-induced wounds were assessed. Desmoglein-1 (DSG-1), a cell adhesion structure playing a role in maintaining the structure of the epidermis through its adhesive function, is located above the basal layer in normal skin^25^. Corneodesmosin (CDSN) is synthesized in lower granular keratinocytes and represents an epidermal basic glycoprotein associated with corneocyte-specific modified desmosomes^26^. In comparison to the expression pattern of DSG-1 and CDSN at the beginning of the culture (Fig. 2A,C), their expression was found in lower epidermal layers at later time points of culture in unwounded skin (Fig. 2B,D). Both DSG1 and CDSN were absent at the beginning of the culture in the wound bed (Fig. 2a,c) but positive in the epidermis of re-epithelialized wounds at day 10 of culture with a similar staining pattern to cultured unwounded control skin (Fig. 2b,d). Filaggrin, which plays a crucial role in skin permeability^27^, was expressed in freshly isolated skin (Fig. 2E) and, apparently more pronounced, at later culture points in unwounded skin biopsies (Fig. 2F), while its expression in the epidermis of re-epithelialized wounds was consistently observed at day 10 after wounding and culture (Fig. 2f).

### Increased frequency of matrix metalloproteinase 9 (MMP-9) expressing cells in wounded skin upon prolonged culture

**Figure 3:**
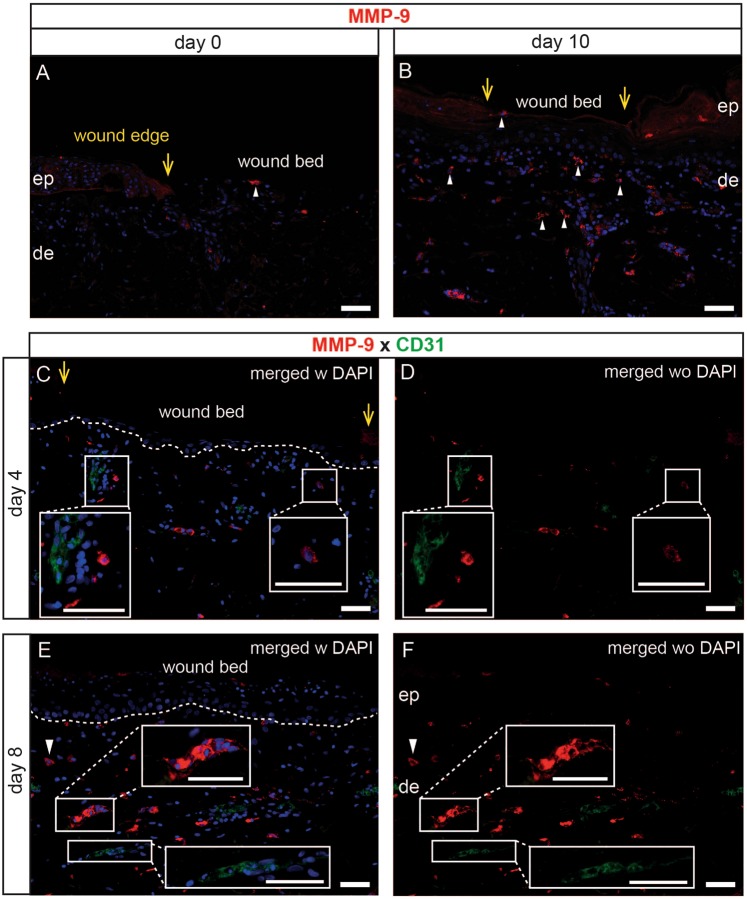
Distinct MMP-9^+^ cells appear with culture. MMP-9^+^ cells are almost absent in freshly wounded biopsies (**A**), while many cell types appear in the epidermis and dermis upon longer culture (**B**–**D**). MMP-9 expression pattern differs in cells. Strong MMP-9 expression is present at wound edges on day 4 and is weaker on day 6 after wounding (**B,C**, upper inserts). In some cells MMP-9 expression appears in dots around cells (**E,F**, right insert), on the cell surface (**G**,**H**, arrowhead) or interconnecting cells forming a ´´vessel-like´´ structure (**G**,**H**, upper insert). Counterstaining with CD31 revealed that dermal cells express either MMP-9 or CD31 (**E,F**, left insert; **G,H**, inserts). Yellow arrows indicate the wound edge. White dotted line indicates the basement membrane. Nuclei are counterstained with DAPI. One representative of three experiments is indicated. ep, epidermis; de, dermis. Scale bars = 50 µm.

MMPs become activated during wound repair. In particular, MMP-9 influences the detachment of basal keratinocytes from the basement membrane thus improving their migration^28,29^. We noticed scarce MMP-9^+^ cells directly under the wound bed and in unwounded skin areas (Fig. 3A). In contrast, strong MMP-9 expression was observed at the leading wound edges at day 4 and already decreased 6 days after wounding (Fig. 3B,C, inserts). MMP-9^+^ cells greatly accumulated in the dermis at day 10 of culture (Fig. 3D, arrowheads). Of note, different MMP-9 expression patterns were observed in dermal cells implying that distinct cell types upregulate this enzyme. MMP-9 appeared in dots around the nucleus (Fig. 3E,F, right insert), on the cell surface (Fig. 3G,H arrowhead) or interconnecting cells forming a “vessel-like” shape (Fig. 3G,H, upper insert). Inspired by the MMP-9 “vessel-like” staining pattern, we tested whether they co-express CD31 which in normal human skin is expressed primarily in endothelial cells lining the interior surface of blood and lymphatic vessels^30^. Surprisingly, we found that dermal cells expressing MMP-9 and CD31 are mutually exclusive, thus excluding their endothelial nature (Fig. 3G,H). The nature of other MMP-9 expressing cells remains to be further explored.

### Negative pressure leads to an influx of dermal cell populations into blister roof epidermis

**Figure 4:**
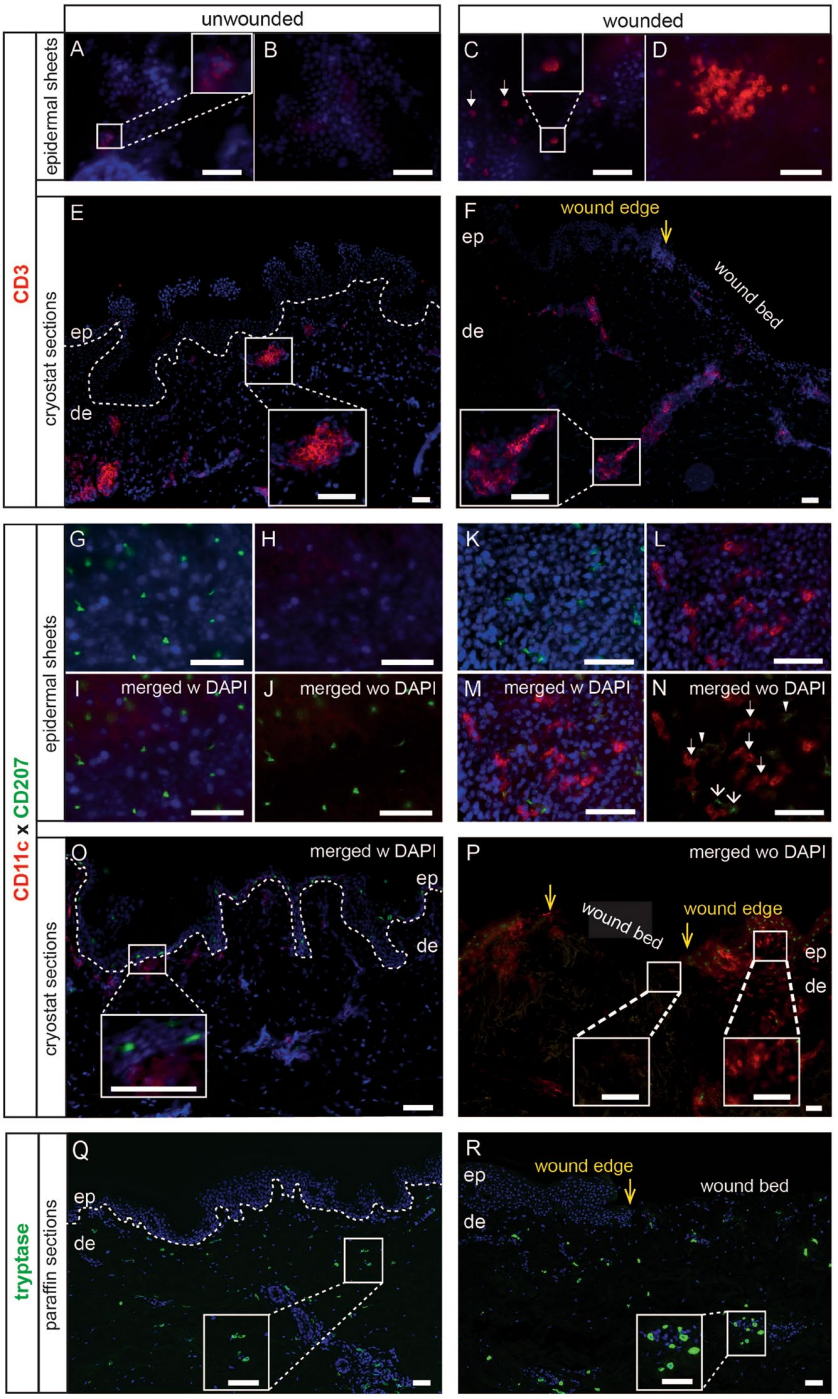
Influx of dermal immune cells into suction blister roof epidermis. A few T cells are visible in unwounded epidermis (**A**, insert; **B**), whereas in blister roof epidermis they are evenly distributed (**C**, arrows, insert) and appear also in clusters (**D**). Immunofluorescence labeling of unwounded sections shows T cells as single cells and clustered in the papillary and reticular dermis (E, insert). In wounded skin, some T cells are located along the wound bed and are consistently less abundant in dermal clusters (**F**, insert) compared to unwounded skin. CD207^+^CD11c^−^ LCs are detected in normal and blister roof epidermal sheets (**G**–**N**), while CD207^−^CD11c^+^ cells with a dendritic morphology are only detected in blister roof epidermis often in close proximity to LCs (**M,N**, open arrows). In unwounded skin, CD207^+^ LCs are distributed throughout the basal and suprabasal epidermal layers and CD11c^+^ DCs are localized mostly in the papillary dermis (**O**, insert). In blister wounded skin, CD207^+^ LCs are absent in the wound bed (**P**). CD11c^+^ DCs are never present in the wounded area as compared to unwounded skin in the same section (**P**, inserts). Tryptase^+^ mast cells were found to be equally distributed throughout dermis and in close proximity to the basement membrane in unwounded controls (**Q**) and wounded sections immediately after wounding (**R**). Yellow arrows indicate the wound edge. Dotted white line demarcates the basement membrane. Nuclei are counterstained with DAPI (blue). One representative of three experiments is demonstrated. ep, epidermis; de, dermis. Scale bars = 50 µm.

The suction blister method has been used in several studies to investigate LCs in human epidermis *in vivo* such as effects of ultraviolet B irradiation^31^, as well as a calcineurin inhibitor and corticosteroids^18^. To determine, whether application of negative pressure affects LCs in *ex vivo*-induced blister roof epidermis, we stained them with markers typically used to identify LCs in human epidermis. We found that suction does not affect their morphology, phenotype and numbers (Fig. S2). In normal human skin, up to 5% of all T cells are present in the epidermis^32,33^. To test whether dermal T cells infiltrate into the blister roof epidermis upon application of negative pressure, normal and blister wounded epidermal sheets as well as skin sections were stained with an Ab directed against CD3. In normal epidermis, only a few, scattered T cells were found (Fig. 4A, insert; B), while many were identified in the blister roof epidermis, some of which were evenly distributed (Fig. 4C) or appeared in clusters (Fig. 4D). This is well mirrored in the dermis where T cells were located in form of clusters or single cells in skin sections of unwounded skin (Fig. 4E), but appeared along the wound bed and less abundant in the dermis (Fig. 4F) when compared to normal skin, implying their partial migration into the epidermis. This finding suggested that also other dermal populations may have infiltrated the epidermis upon negative pressure and/or markers may have been upregulated on resident LCs. Confirming our previous observation in normal skin^34^, CD11c expression was undetectable on resident LCs in normal epidermis (Fig. 4G–J) and blister roof epidermis (Fig. 4K-N). Surprisingly, CD11c^+^ cells with a polygonal to dendritic shape were abundantly present in the blister roof epidermis as a separate population (Fig. 4K–N). Our further observation that CD11c^+^ cells were present in the unwounded dermis (Fig. 4P, right insert) but were absent in the blister wounded dermis underneath the wound bed in the same section (Fig. 4P, left insert) support the validity of our assumption about their dermal derivation. In contrast to T cells and DCs, we found mast cells with a comparable morphology and distribution in unwounded and wounded dermal areas in skin biopsy sections (Fig. 4R) as compared to unwounded controls (Fig. 4Q), suggesting that their localization is not influenced upon suction.

**Figure 5:**
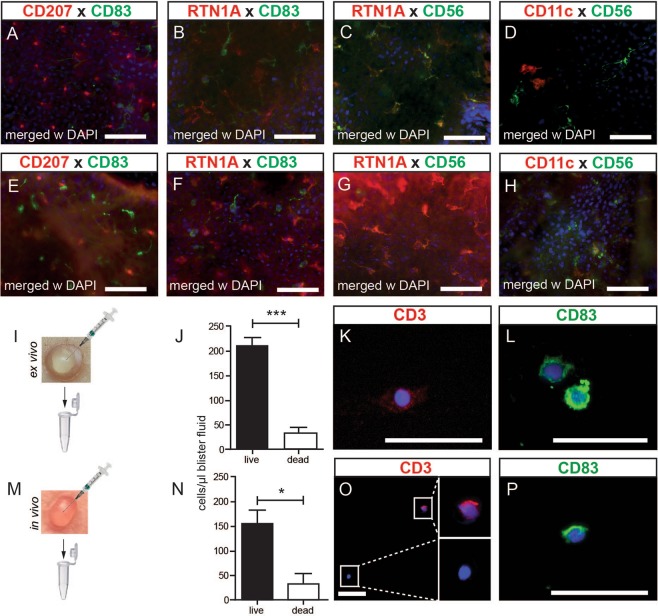
Identification of viable immune cells in suction blister fluid. CD83^+^ cells in blister roof epidermis *ex vivo* (**A**–**D**) and *in vivo* (**E**–**H**) do not co-express CD207 (**A,E**) and RTNA1 (**B,F**). CD56^+^ cells co-express either RTN1A (**C,G**) or CD11c (**H**) or are single positive (**C,D,G,H**). Cell numbers were counted from *ex vivo* (**I**) and *in vivo* (**M**) induced suction blisters. Dead cells were determined by trypan blue exclusion (**J**, **N**; n = 3). Bars represent mean ± SD of investigated groups. Paired student t-test was used. *P < 0.05, ***P < 0.001. Immunofluorescence labeling shows CD3^+^ and CD83^+^ cells on adhesion slides obtained from *ex vivo* (**K,L**) and *in vivo* (**O,P**) suction blister fluid. Nuclei are counterstained with DAPI. One representative of three experiments is demonstrated. Scale bars = 50 µm.

Next, the activation marker CD83 was used to further characterize the CD11c^+^ cell population in blister roof epidermis. While CD83^+^ cells were indeed found in the blister roof epidermis, they never co-expressed CD207 (Fig. 5A; Fig. S3E–H), CD11c (Fig. S3M–P) and RTN1A, a recently identified marker for cells of the DC lineage^34^ (Fig. 5B; Fig. S3U-X). These data show that neither LCs nor CD11c^+^ cells are activated and demonstrate the presence of an additional cell population in blister roof epidermis. The absence of CD83^+^ cells in normal epidermis (Fig. S3A–D,I–L,Q–T) suggests that they derive from the dermis, or alternatively, that CD83 is upregulated on a dendritically shaped epidermal population. Even though CD83 was identified recently also as a marker for a unique T cell population^35^, the dendritic appearance of CD83^+^ cells is not in favour of a T cell nature. The presence of cell populations in *ex vivo* blister roof epidermis, usually not identified in the epidermis, was intriguing. To test whether this observation is an *in vitro* phenomenon or occurs *in vivo* as well, we investigated blister roof epidermis obtained from healthy volunteers. Again, CD83^+^ cells were observed that never co-expressed CD207 (Fig. 5E; Fig. S4a–d) or RTN1A (Fig. 5F; Fig. S4e–h).

To test whether the dendritically shaped cells may belong to the nerve network in human skin, we employed an antibody directed against the neural cell adhesion molecule-1 (NCAM-1)/CD56^36^. In the dermis, terminal nerve endings are enveloped by single CD56^+^ Schwann cells^37^. Schwann cells constitute a substantial cell population at dermo-epidermal junctions, where the expression of several markers, including CD56, on myelinating cells was found only in the dermis and not in peripheral nerves^38^. Although mainly considered as a marker of neural lineage, more recently, expression of CD56 on natural killer cells, γδ T cells, activated CD8^+^ T cells and DCs has been reported^39^. As expected, no CD56^+^ cells were found in normal epidermis (Fig. S4A–D,I–L). Interestingly, the majority of RTN1A^+^ cells co-expressed CD56 (Fig. 5C; Fig. S4E–H) but never CD11c (Fig. 5D; Fig. S4M–P) in blister roof epidermis. Besides CD56/RTN1A double positive cells, we identified cells expressing either of the two markers in blister roof epidermis (Fig. 5C; Fig. S4E–H). These observations suggest that all distinct populations described in blister roof epidermis most likely derive from the dermis. Similar populations were observed in blister roof epidermis obtained from healthy volunteers *in vivo* (Fig. 5G; Fig. S4i–l). Remarkably, and in addition to CD56^+^CD11c^−^ and CD56^−^CD11c^+^ cells, also CD56^+^CD11c^+^ cells were present (Fig. 5H; Fig. S4m–p). As mast cells remain in the dermis upon suction (Fig. 4R), it is highly unlikely that some of aforementioned cells in the epidermis are mast cells.

### Viable immune cells are present in suction blister fluid

To test the validity of our assumption that distinct populations in the blister roof epidermis may be derived from the dermis, we tested *ex vivo* and *in vivo* blister fluids (Fig. 5I,M). In a first step, we enumerated all cells and found that the frequency of live cells as compared to dead cells was always significantly higher (Fig. 5J,N). Staining of these cells on adhesion slides revealed CD3^+^ cells (Fig. 5K,O) and CD83^+^ cells (Fig. 5L,P), suggesting their derivation from the dermis.

## Discussion

Many skin wound healing studies in humans were conducted with *ex vivo* models^9,10,40^. To our knowledge, we have efficaciously applied for the first time negative pressure (suction) to *ex vivo* human skin leading to the formation of typical blisters. Of note, for all experiments shown in the manuscript we have used abdominal skin only. We would like to emphasize that similar results were repeatedly obtained when suction was applied on *ex vivo* thigh and arm skin, showing that the method is reproducible irrespective of the body location which is important as abdominal skin is not always available. Upon removal of the blister roof, we found neither damage of Col-IV nor epidermal components on the suction blister floor (=wound bed) which is in line with several studies showing by electron microscopic and histochemical analysis that the basement membrane stays intact after suction blister formation^14,20,22^. The absence of keratinocytes in the initial wound bed implied that keratinocytes have migrated and proliferated from the wound edges across the wound bed to close the wound upon culture. Indeed, Ki67^+^ cells were found along the initial wound bed in the basal layer upon re-epithelialization within 6 days, or in some cases, even within 4 days (data not shown). This was somewhat delayed compared to *in vivo* experiments^41^ and may be due to a deficiency of factors under culture conditions that are produced by newly arriving cells from the circulation *in vivo* and/or removal of the dermal cells. Nevertheless, upon prolongation of culture, epidermal cells differentiated and expression of K10 by basal keratinocytes was only found at the wound edges 8 days after wounding, which was hypothesized to be due to quick rolling of already differentiated keratinocytes on top of the wound bed which did not degrade suprabasal keratins^42^. No K10^+^ keratinocytes were found along the basement membrane at later time points. Decreasing expression of K10 was found in suprabasal keratinocytes with time of culture and is in line with observations in other human wound model studies^2,43,44^. Both DSG1 and CDSN were expressed 6 days post wounding already above the initial wound bed and persisted until the end of the culture at day 12. Their formation was observed in lower epidermal layers in blister-induced wounds only and does not occur in normal skin, suggesting a potential premature cornification, together with the increased thickness of the *stratum corneum*, persistence of nuclei and possibly reduced thickness of spinous and granular layers. Filaggrin was expressed above the former wound bed 10 days post wounding, suggesting a re-establishment of a “normal” differentiation pathway. Our observation that the *stratum corneum* in unwounded skin was obviously conserved and occasionally increased upon prolonged culture, is in line with another study, suggesting that we used the adequate air-liquid interphase for our culture system, which allows keratinocytes to form the *stratum corneum*^9^. Taken together, suction blister-induced wounds obtained *ex vivo* have the characteristics of the regenerative pathway, which makes them suitable to study wound healing in terms of (i) re-epithelialization and (ii) therapeutics and their targets.

Cytokines and in particular MMP-9 play a major role during wound repair. The MMP-9 expression pattern at the wound edges and its substrate preference for the basement membrane is thought to be responsible for detaching basal keratinocytes from the basement membrane, thereby promoting their migration and wound closure^28,29,45^. We found strong MMP-9 expression at wound edges, which confirms and extends previous findings by Hattori and colleagues showing expression of MMP-9 at the leading edges of epidermal keratinocytes of wounded wild-type mice. MMPs are present in both chronic and acute wounds and their activity must be controlled at all stages of wound healing process for successful wound closure^46^. It has been shown that human mast cells constitutively produce MMP-9^47^, which in the dermis is crucial for the degradation of extracellular matrix^48^. In line with this, we found dermal cells expressing MMP-9 in form of small dots, highly suggesting that these cells are activated de-granulated mast cells^49^.

We further observed MMP-9^+^ cells with a typical vessel like shape in our blister-induced wounds upon culture. MMP-9 has been reported to increase VEGF release and to promote angiogenesis^50^ and expression of MMP-9 by endothelial cells has been shown to be one of the main prerequisites for successful angiogenesis^51^. In our suction blister-induced wound model, however, all MMP-9^+^ cells with a typical “vessel-like” shape in the dermis did not co-express CD31 and could be explained by a lacking blood supply in our model.

Together with keratinocytes, also LCs are exposed to negative pressure in our *ex vivo* model. Interestingly, LCs were not affected in terms of their morphology, phenotype and numbers. This is consistent with *in vivo* induced blisters when the influence of drugs was tested on LCs^18^. While the suction did not affect LCs in the blister roofs, we found, to our big surprise, the presence of evidently increased T cell numbers in blister roof epidermis, as compared to the rare population in normal epidermis. Many studies in humans have used induction of blistering and collection of suction blister fluid for analysis *in vivo* to (i) investigate cutaneous T cell recall responses^52,53^, and (ii) study T cells from toxic epidermal necrolysis patients^54^. Our finding that T cells can be identified in suction blisters induced *in vitro* confirms and expands previous studies and suggest their potential immigration into the epidermis. Our further data that CD207^−^CD83^+^ cells with a dendritic morphology were identified not only in the epidermis but also in the suction blister fluid, imply that they are presumably of dermal origin, able to move through the suction blister fluid and infiltrate into the suction blister roof by mechanical application of negative pressure. Staining with more markers and advanced microscopy techniques are necessary to better characterize the identified cell populations in blister roof epidermis.

Topical negative pressure is applied in clinical practice in the management of difficult wounds. It is described as short, safe and simple treatment, beneficial to wound healing by increasing local blood flow, reducing bacterial colonization rates and removing excess wound exudates thereby providing the ideal wound healing environment^55^. Our finding of living cells in *ex vivo* as well as *in vivo* suction blister fluid is intriguing. It is conceivable that removal of immune cells from the wound bed, and consequently secretion of related factors, might be an additional explanation for the beneficial effect of this method for the wound healing process including a more rapid re-epithelialization *in vivo* and remains to be further investigated.

Our *ex vivo* method poses a few limitations, including variable wound size due to the unequal blister formation. Furthermore, the removal of dermal cells by the suction blistering process and/or a lack of newly incoming cells from the circulation might cause a slight delay of re-epithelialization. The prolonged suction time to induce blister formation *ex vivo* compared to the *in vivo* situation and the need of higher pressure might be due to the lack of tension and backpressure of the body and/or the lacking blood flow. Addressing these questions is not trivial and will need more sophisticated experiments in the future.

In conclusion, our study contributes to the current knowledge on blister technology *in vivo* by showing for the first time that also *ex vivo* suction blistering on human skin is feasible, thereby generating standardized wounds, blister roofs and suction blister fluid, all of which could be used for different purposes in dermatological and immunological research. With regard to re-epithelialization of untreated standardized wounds, we showed that this model recapitulates the main features of human epidermal regeneration and might be useful in the future to study the efficacy of drugs in small standardized wounds in humans.

## Methods

### Ethics statement

Healthy skin was obtained from routine reduction surgeries from anonymous donors and experiments on *ex vivo* skin were typically performed within 1–2 hours (h) after surgery. The study was approved by the ethics committee of the Medical University of Vienna and conducted according to the Declaration of Helsinki principles. Written informed consent from all participants was obtained.

### Suction blister-induced wound model

A negative pressure instrument (Electronic Diversities, Finksburg, MD, USA) constructed to produce standard suction blisters upon application of negative pressure, was used on healthy skin (*ex vivo:* abdominal skin; *in vivo:* lower forearm). Subcutaneous fat was partially removed from *ex vivo* skin using a scissor. Subsequently, skin (10 × 10 cm^2^) was placed (not fixed, not kept in medium) on a styrofoam lid that was covered with aluminium foil to provide (at least partial) backpressure. Suction chambers with 5 openings (Ø = 5 mm) on the orifice plate were attached to skin, topped with a styrofoam lid and pressed with 1 kg weight in order to avoid movement of the plate. A pressure of 200–250 millimeter (mm) mercury (Hg) (*ex vivo*) or 150–200 mm Hg (*in vivo*) caused the skin to be drawn through the openings creating typical suction blisters of different size within 6–8 h (*ex vivo*) and 1–2 h (*in vivo*). Suction blister fluid (~110 µl/5 blisters) was collected using a syringe with a needle. Cells within the fluid were counted and placed on adhesion slides for staining and analysis. Blister roof epidermis was cut with a scissor, fixed with ice-cold acetone (10 minutes) and used for staining. For comparison and control, epidermal sheets were prepared from unwounded skin biopsy punches (Ø = 6 mm; 3.8% ammonium thiocyanate (Carl Roth GmbH + Co. KG, Germany) in PBS (Gibco, Thermo Fisher, Waltham, MA, USA), 1 h, 37 °C). Removal of the blister roof created a wound area. Biopsies (Ø = 6 mm) from wounded and unwounded areas were cultivated for 12 days in either duplicates or triplicates in 12 well culture plates and Dulbecco’s modified Eagle’s medium (DMEM) (Gibco) supplemented with 10% fetal bovine serum (FBS) (Gibco) and 1% penicillin-streptomycin (Gibco) and were cultured at the air-liquid interphase. Medium was changed every second day.

### Hematoxilin and eosin (H&E) staining

Freshly isolated and cultivated skin samples were harvested at time points, as indicated in the figures, and fixed immediately in 7.5% paraformaldehyde (SAV Liquid Production, Flintsbach am Inn, Germany). Fixed tissues were embedded in paraffin, sectioned (5 µm) (Microtome HM 335 E–Microm, GMI, USA) and stained with H&E solution according to standardized protocols.

### Immunofluorescent staining

Freshly isolated and cultivated skin samples were harvested at indicated time-points, embedded in optimum cutting tissue compound (Tissue-plus; Scigen Scientific, Gardena, CA, USA), snap frozen in liquid nitrogen and stored at −80 °C until further processing. Frozen tissues were sectioned (5 µm) (Cryotome–Leica Biosystems CM1850, Germany), fixed in ice-cold acetone (10 minutes) and washed with PBS. Fixed sections were stained with unconjugated and conjugated antibodies (Abs) (overnight, 4 °C) and Ab binding was detected using corresponding secondary Abs. Paraffin embedded tissues were deparaffinised by dipping them into Xylol (2x, 5 minutes), 100% ethanol (5 minutes), 70% ethanol (5 minutes) and washed in tap water (2x, 5 minutes). Then they were incubated in antigen retrieval buffer (Dako S1699, Denmark), washed in PBS and stained. Abs used are listed in Table S1.

### Statistical analysis

Data was analysed using GraphPad Prism 5 (GraphPad Software, La Jolla, CA, USA). Paired t-test was used for comparing means. The results were considered significant with *P*-values smaller than 0.05.

## Supplementary information

Supplementary Information

## Data Availability

Data, in anonymous format (according to data protection policy in the ethics agreement) is available on reasonable request.
