# Supplementary material for: Re-epithelialization and immune cell behaviour in an ex vivo human skin model
Source: Sci Rep. 2020 Jan 8;10:1. doi: 10.1038/s41598-019-56847-4 (PMC6959339; doi:10.1038/s41598-019-56847-4)
Supplement: Supplementary file 1 — Supplementary Information [file 41598_2019_56847_MOESM1_ESM.docx]

# Supplementary information

# Re-epithelialization and immune cell behaviour in an *ex vivo* human skin model

# Ana Rakita, Nenad Nikolic, Michael Mildner, Johannes Matiasek, Adelheid Elbe-Bürger

**Content**

Supplementary Table 1

Supplementary Figures S1-S4

**Supplementary Table S1. Abs used for immunofluorescent staining.**

| **Primary Abs** | **Sample type** | **Dilution** | **Company & Species** | **Secondary Abs** |
| --- | --- | --- | --- | --- |
| Collagen type IV | Cryo section | 1:500 | Abcam, ab6586  rabbit polyclonal Ab | Life Technologies  goat anti-rabbit Alexa Fluor 546 |
| Ki67 | Cryo section | 1:400 | Cell Signaling, D3B5  rabbit monoclonal Ab | Life Technologies  goat anti-rabbit Alexa Fluor 488 |
| K14 | Paraffin section | 1:400 | ThermoFisher Scientific, LL002  mouse monoclonal Ab | Life Technologies  goat anti-mouse Alexa Fluor 546 |
| K10 | Paraffin section | 1:500 | Abcam, ab9025  mouse monoclonal Ab | Life Technologies  goat anti-mouse Alexa Fluor 546 |
| Corneodesmosin  (CDSN) | Paraffin section | 1:100 | Abcam, 204235  rabbit polyclonal Ab | Life Technologies  goat anti-rabbit Alexa Fluor 546 |
| Desmoglein-1  (DSG-1) | Paraffin section | 1:500 | Abcam, 124789  rabbit polyclonal Ab | Life Technologies  goat anti-rabbit Alexa Fluor 546 |
| Filaggrin | Paraffin section | 1:1000 | Covance, 417-P  rabbit polyclonal Ab | Life Technologies  goat anti-rabbit Alexa Fluor 546 |
| MMP-9 | Paraffin section | 1:400 | Abcam, ab76003  rabbit, monoclonal Ab | Life Technologies  goat anti-rabbit Alexa Fluor 546 |
| CD31 | Paraffin section | 1:100 | Dako, #M0823  mouse monoclonal Ab | Life Technologies  goat anti-mouse Alexa Fluor 488 |
| CD207-Alexa488 | Cryostat section, epidermal sheet | 1:200 | Dendritics, 929F3.01  rat monoclonal Ab |  |
| CD207 | Cryostat section, epidermal sheet | 1:400 | Sigma Aldrich, MPA 011 216 rabbit Ab | Life Technologies  goat anti-rabbit Alexa Fluor 488/546 |
| CD1a-FITC | Epidermal sheet | 1:50 | BD Pharmingen, 3037965  mouse Ab |  |
| HLA-DR-Alexa488 | Epidermal sheet | 1:50 | Biolegend, mIgG2 |  |
| CD3-PE | Cryostat section,  suction blister fluid, epidermal sheet | 1:50 | BD Biosciences, 345765 |  |
| CD11c-PE | Cryostat section, epidermal sheet | 1:12.5 | BD Biosciences, 333-149 |  |
| RTN1A | Epidermal sheet | 1:400 | Sigma, A044249  rabbit, HP | Life Technologies  goat anti-rabbit Alexa Fluor 546 |
| CD83 | Epidermal sheet, suction blister fluid | 1:200 | BD Pharmingen, 556854  mouse Ab | Life Technologies  goat anti-mouse Alexa Fluor 488 |
| CD45-FITC | Epidermal cell suspension | 1:50 | Miltenyi Biotec, REA747 |  |
| CD56 | Epidermal sheet | 1:400 | R&D Systems, 301021  mouse monoclonal Ab | Life Technologies  goat anti-mouse Alexa Fluor 488 |
| Tryptase | Paraffin section | 1:1500 | Abcam, ab2378  mouse monoclonal Ab | Life Technologies  goat anti-mouse Alexa Fluor 488 |

**
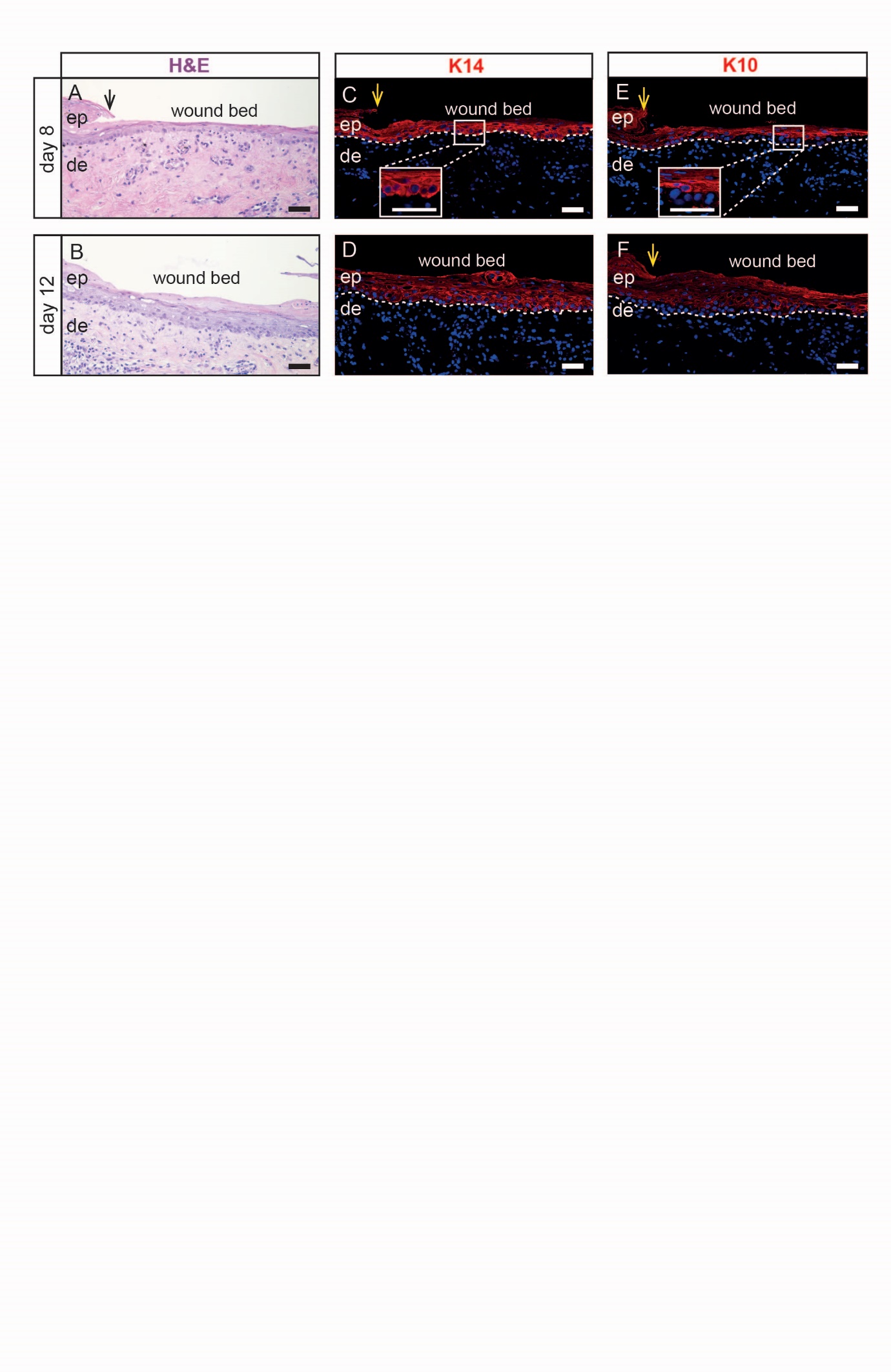
**

**Suppl. Fig. S1. Re-epithelialization kinetics of *ex vivo* suction blister-induced wounds.** Hematoxylin and eosin (A, B) as well as immunofluorescence staining (C-F) of paraffin skin sections shows increasing K14^+^ and K10^+^ keratinocyte layers during the culture period of 12 days. A strong K14 staining of the basal and suprabasal layers is observed at day 8 of culture (C) and appears across all layers with prolonged culture duration (D). K10 is expressed in basal keratinocytes at the wound edge and in suprabasal keratinocytes in the middle of the initial wound bed on day 8 of culture (E). At day 12 of culture K10 expression is observed in all layers with exception of basal keratinocytes (F). Black and yellow arrows indicate the wound edge. White dotted line indicates the basement membrane. Nuclei are counterstained with DAPI. One representative of three experiments is demonstrated. ep, epidermis; de, dermis. Scale bars = 50 µm.


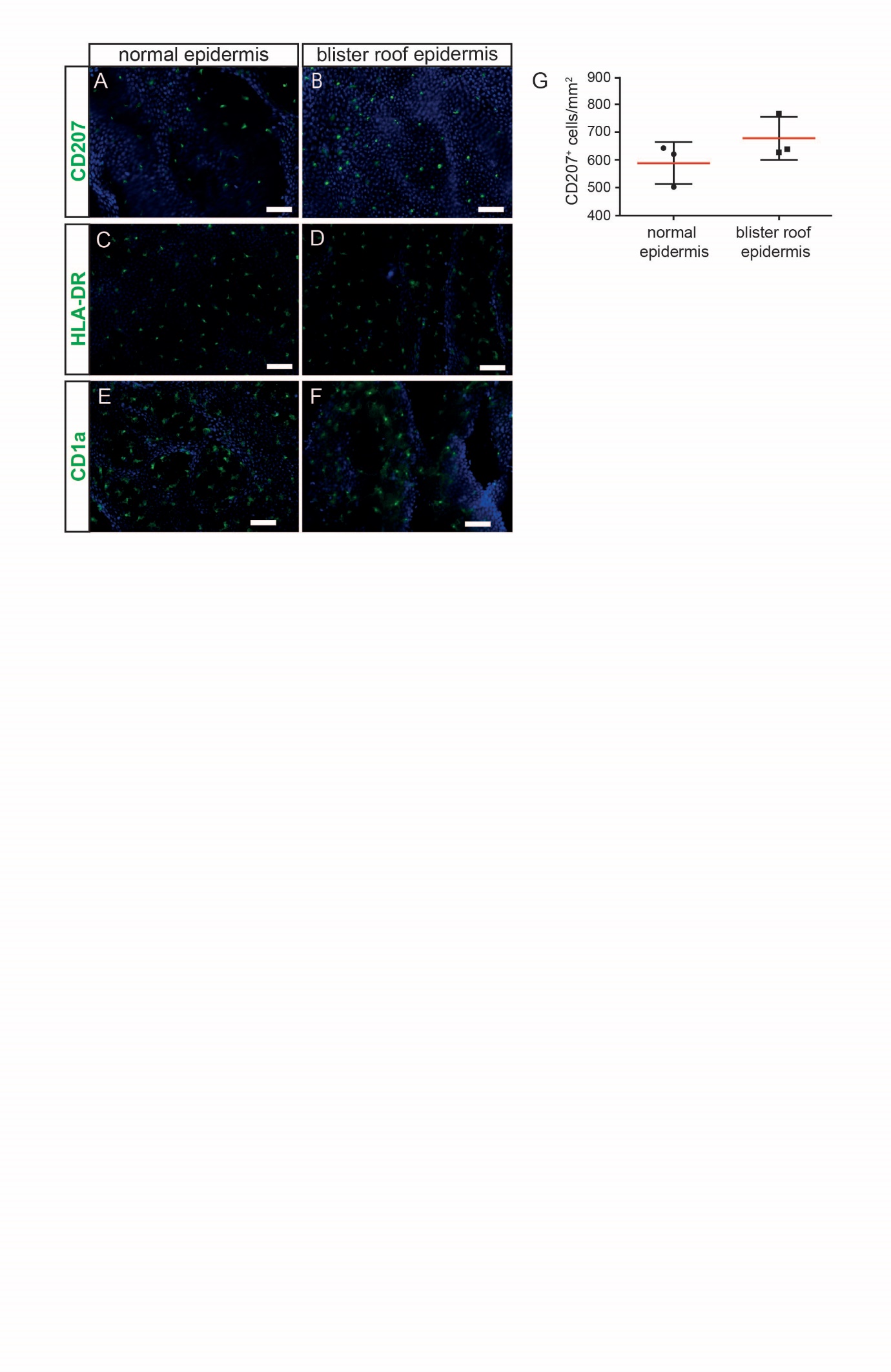


**Suppl. Fig. S2. Negative pressure does not affect morphology, phenotype and density of LCs.** Immunofluorescence staining with indicated markers shows no obvious alterations of LCs in blister roof epidermis (B, D, F) compared to normal epidermis (A, C, E). Representative images of three experiments are shown. Nuclei are counterstained with DAPI. LC density was evaluated by counting five different areas of one epidermal sheet from three different donors (G). Bars represent mean ± SD of investigated groups. Paired student-t test is used and no significant differences were observed.
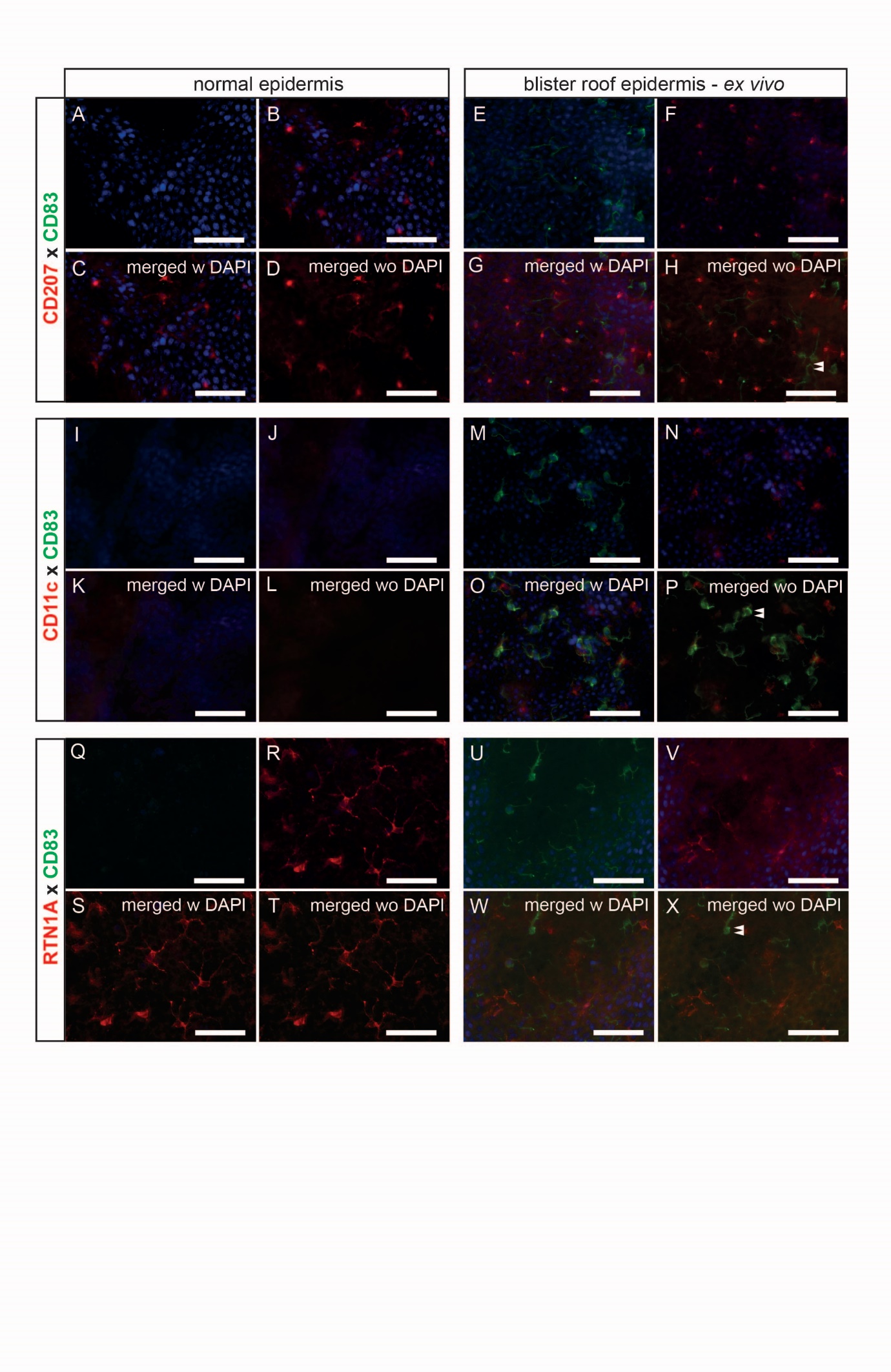
Scale bars = 50 µm.

**Suppl. Fig. S3. CD83^+^ cells in blister roof epidermis do not co-express typical DC markers.** CD83^+^ cells are undetectable in normal epidermal sheets (A-D, I-L, Q-T), but are: present in blister roof epidermis, dendritic in shape, in close vicinity to CD207^+^, CD11c^+^ and RTN1A^+^ cells, and never co-express CD207 (E-H, double arrowhead), CD11c (M-P, double arrowhead) or RTN1A (U-X, double arrowhead). Nuclei are counterstained with DAPI. One representative of three experiments is presented. Scale bars = 50 µm.


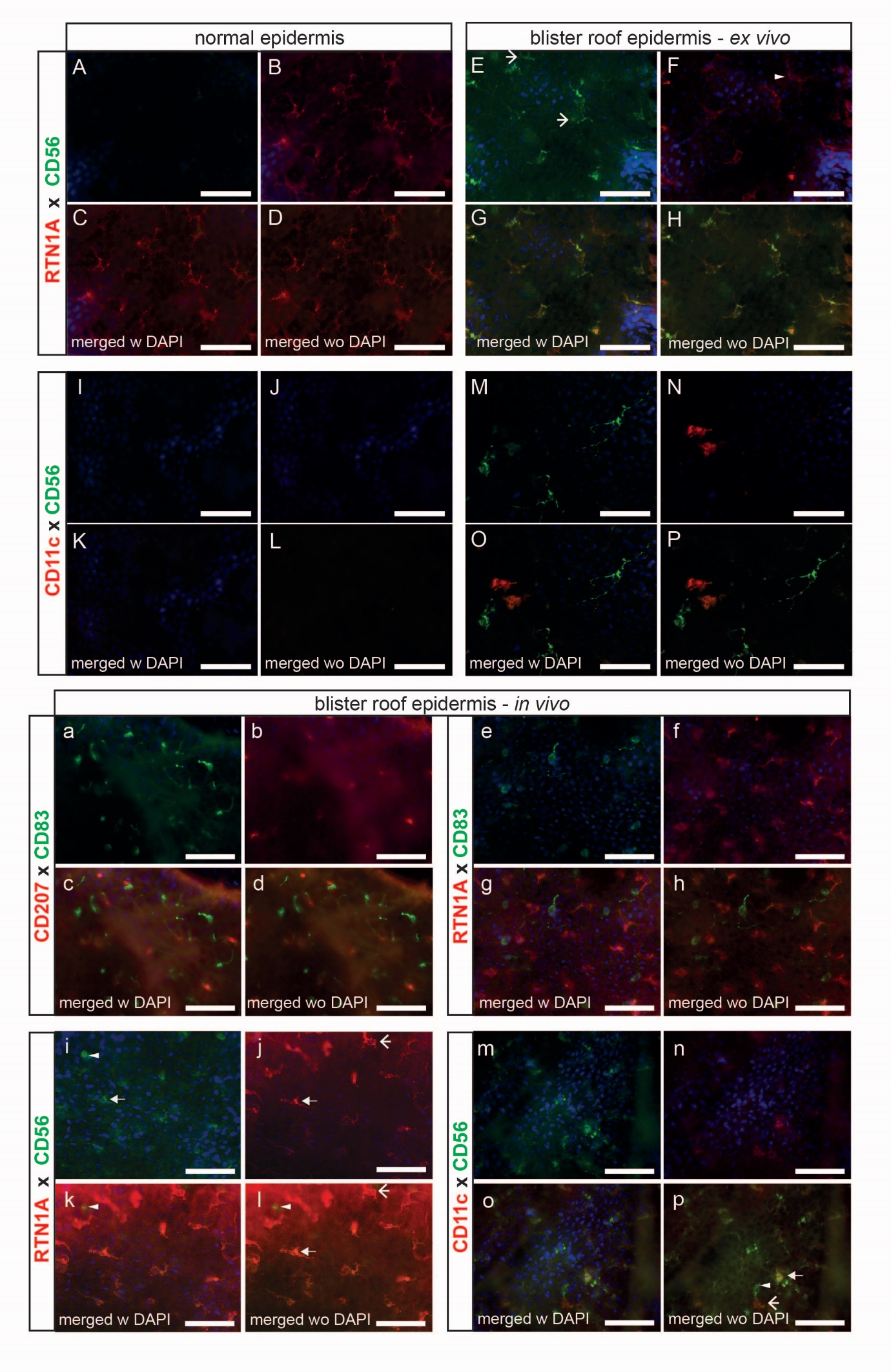


**Suppl. Fig. S4. Cell populations absent in normal epidermis are present in blister roof epidermis.** Immunofluorescent staining showed no CD56^+^ cells in normal epidermis (A-D, I-L). The majority of CD56^+^ cells found in blister roof epidermis *ex vivo* co-expressed RTN1A (G, H). A few cells were single positive (E, F, open arrows, arrowhead). CD56^+^ cells did not co-express CD11c *ex vivo* (M-P). In blister roof epidermis obtained *in vivo*, CD83^+^ cells did not co-express CD207 (a-d) or RTNA1 (e-h). Staining with antibodies directed against CD56 and RTN1A revealed that the majority of cells were double positive (i, j, l, arrow). Some cells expressed either RTN1A (j, l, open arrow) or CD56 (i, k, l, arrowhead). Most of the CD56^+^ cells did not co-express CD11c (m-p). Nuclei are counterstained with DAPI. Representative image of two experiments is shown. Scale bars = 50 µm.
